# Acute Unilateral Blindness from Superior Ophthalmic Vein Thrombosis: A Rare Presentation of Nephrotic Syndrome from Class IV Lupus Nephritis in the Absence of Antiphospholipid or Anticardiolipin Syndrome

**DOI:** 10.1155/2015/413975

**Published:** 2015-12-21

**Authors:** Firas Baidoun, Rommy Issa, Robert Ali, Bashar Al-Turk

**Affiliations:** Internal Medicine Department, University of Florida College of Medicine, Jacksonville, FL 32209, USA

## Abstract

Patients with systemic lupus erythematosus (SLE) are at high risk of arterial and venous thrombosis secondary to anti-phospholipid antibodies. Herein, we are presenting an interesting case of venous thrombosis in a patient with SLE in the absence of anti-phospholipid antibodies.

## 1. Introduction

Superior ophthalmic vein thrombosis as a result of nephrotic range proteinuria from lupus nephritis in the absence of antiphospholipid syndrome is a very rare entity and there is paucity of research on this topic in the literature. However, this phenomenon is a medical emergency and warrants immediate recognition and treatment to prevent permanent blindness.

## 2. Case Report

**Figure 1:**
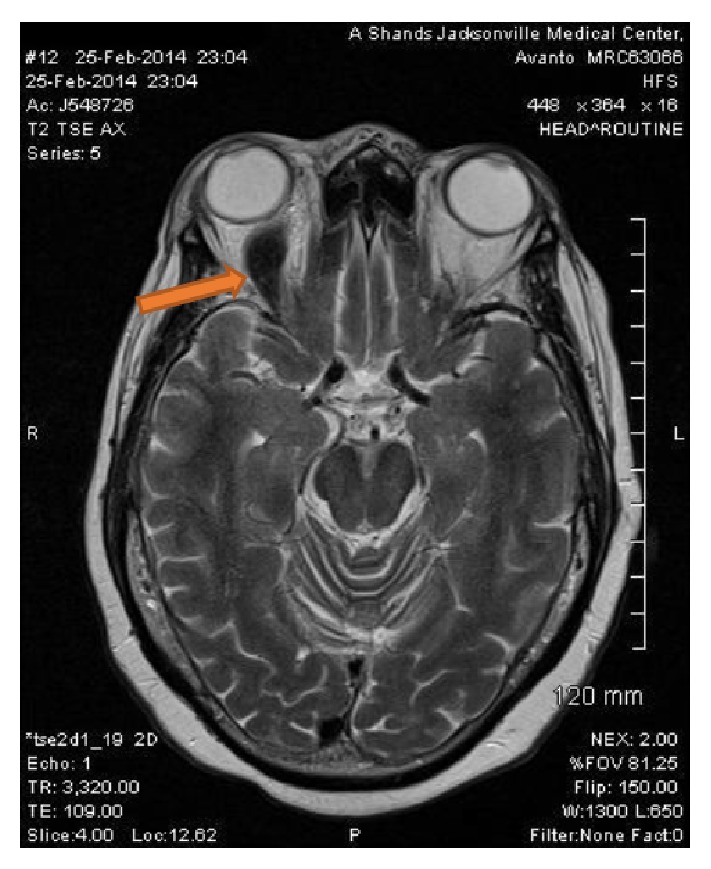
Brain MRI. Axial section (T2) at initial presentation. The arrow is pointing to decreased signal within right superior ophthalmic vein thrombosis.

**Figure 2:**
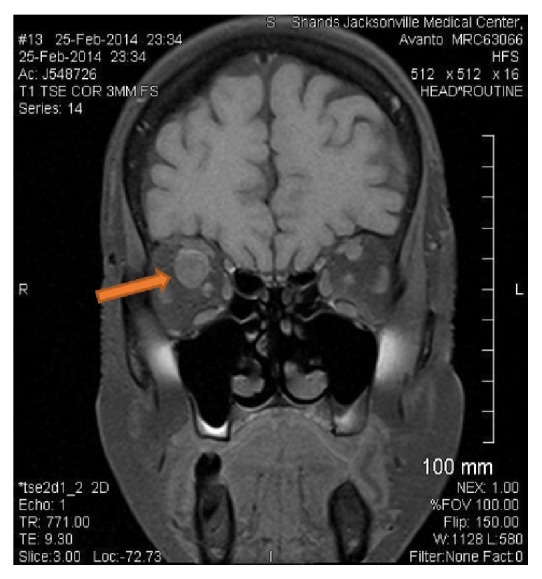
Brain MRI. Coronal section (T1) at initial presentation. The arrow is pointing to right SOV thrombosis.

**Figure 3:**
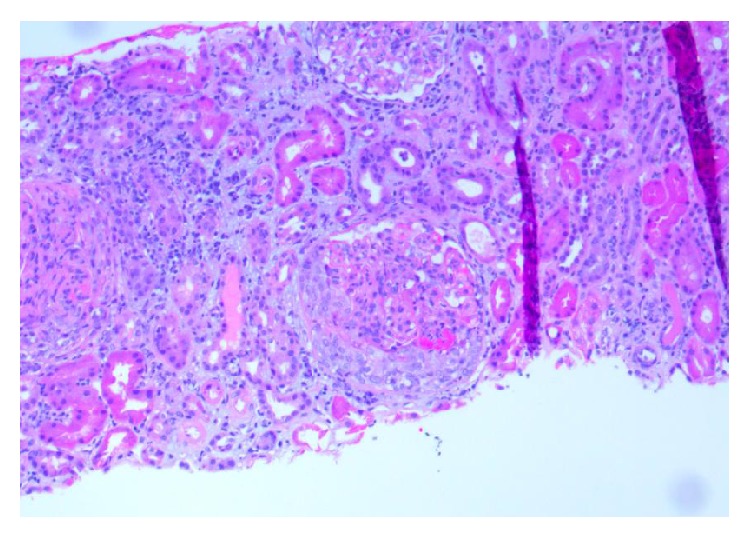
Section of kidney biopsy with increased endocapillary cellularity, cellular crescent, and chronic interstitial inflammation (H&E 10x).

**Figure 4 fig4:**
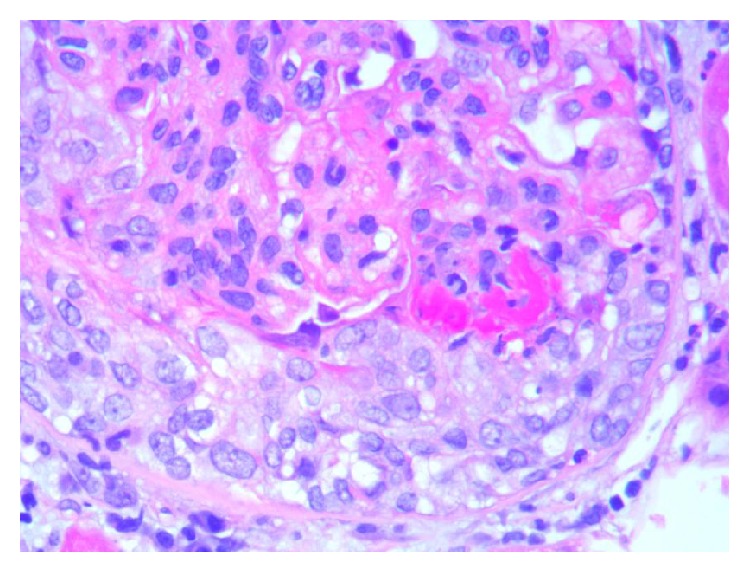
High power of kidney biopsy with cellular crescent, glomerular leukocyte infiltration, and fibrinoid necrosis (H&E 40x).

**Figure 5 fig5:**
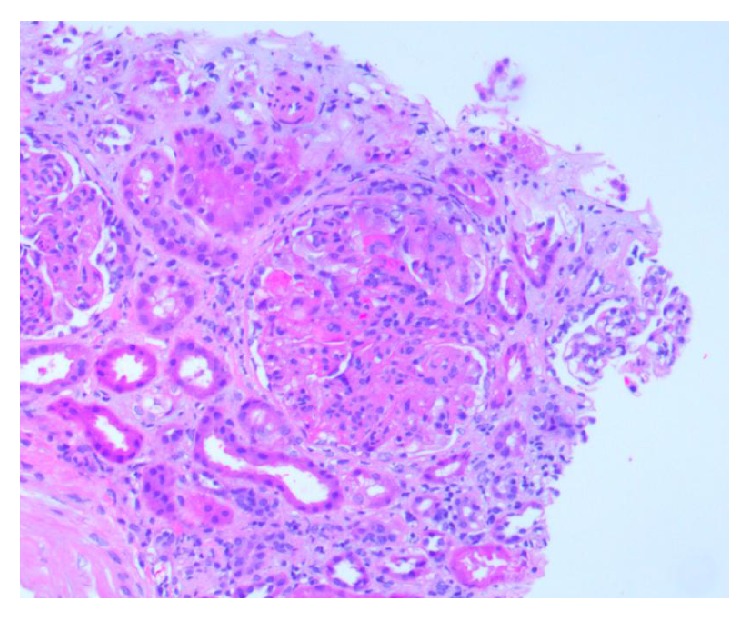
Low power view of kidney biopsy with increased mesangial matrix and endocapillary cellularity and chronic interstitial inflammation (H&E 10x).

**Figure 6 fig6:**
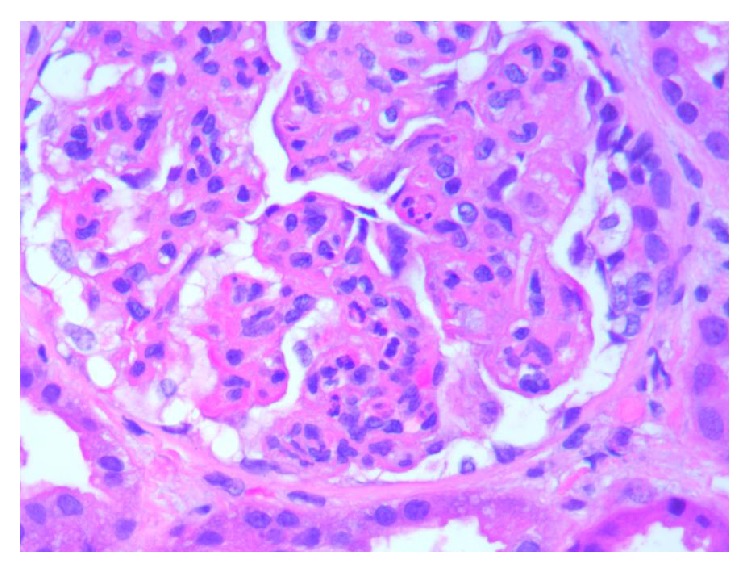
High power view of kidney biopsy with increased mesangial matrix, endocapillary proliferation, and glomerular leukocyte infiltration (H&E 40x).

**Figure 7:**
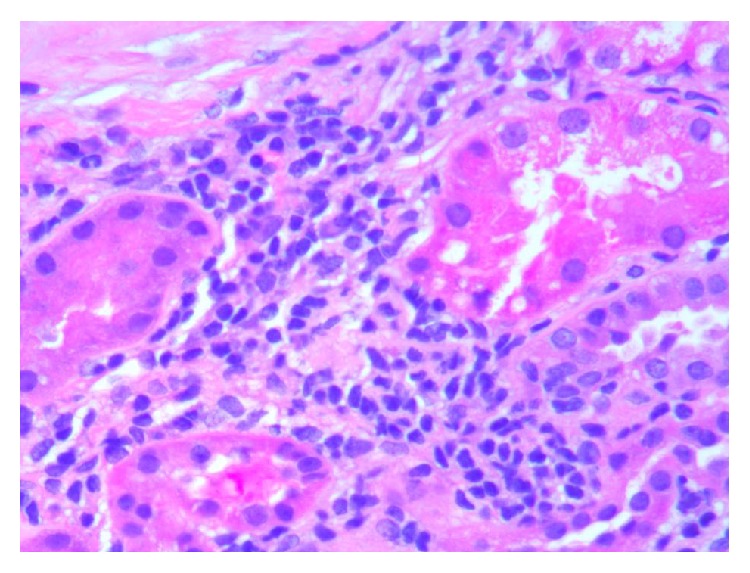
High power view of kidney biopsy with tubular atrophy and chronic interstitial inflammation composed of lymphocytes and plasma cells (H&E 40x).

The patient under discussion is an African American female in her 60s with no significant past medical history, who presented to the Emergency Department with abrupt right eye blindness. She had been postmenopausal for several years, had not been on contraceptive agents, and was never a smoker. She had no known medical history of previous venous thromboembolism and had no known malignancy or coagulopathy. Her medical history was also unremarkable for renal, cardiac, or hepatic diseases. Initial workup showed a creatinine level of 4.0 and a urine protein-to-creatinine ratio of 16. Protein S and antithrombin III levels were reduced to 38% (normal range: 70 to 140) and 65% (normal range: 80 to 120), respectively, whereas protein C activity was within normal limits. MRI of the orbits revealed occlusive superior ophthalmic vein thrombosis (Figures 1 and 2). Patient was treated with hemodialysis for anuria and a renal biopsy was pursued to determine the cause of her nephrotic syndrome. The biopsy results revealed class IV lupus nephritis (Figures 3 [Fig fig4] [Fig fig5] [Fig fig6]–7). Her immunological profile was remarkable for positive ANA (antinuclear antibody), anti-Smith, and anti-ds DNA along with anti-Ro/SSA autoantibodies. C3 and C4 complement levels were not decreased. She did not have anti-phospholipid and anti-cardiolipin antibodies. Anti-neutrophil cytoplasmic antibodies and anti-glomerular basement membrane antibody were both unremarkable. Relevant hematological workup was also noncontributory. Class IV lupus nephritis and secondary Sjögren's syndrome were treated with monthly doses of intravenous cyclophosphamide with twice daily doses of hydroxychloroquine 200 mg and pulse dose steroids. However, she developed hemorrhagic cystitis from cyclophosphamide and it was switched to Mycophenolate Mofetil. Superior ophthalmic vein thrombosis was treated with heparin bridged with warfarin to a goal INR between 2 and 3. While her proteinuria improved, the onset of hemorrhagic cystitis in setting SOVT posed a significant management issue. After a brief initial hold, warfarin was continued through her lengthy episode of hematuria and she was switched to 1 gram twice daily regimen of Mycophenolate Mofetil. Eventually, the patient recovered with complete return of her vision and substantial improvement in proteinuria to less than 2 grams per 24 hours. Unfortunately, due to the underlying fibrosis from lupus nephritis, her renal function did not improve and she continued to receive hemodialysis treatment at the time of her discharge from the hospital.

## 3. Discussion

Acute unilateral blindness is a medical emergency with severe medicolegal repercussions. Superior ophthalmic vein thrombosis from nephrotic syndrome in the setting of newly diagnosed class IV lupus nephritis in the absence of antiphospholipid syndrome is a unique phenomenon. The cause of this thrombosis was the hypercoagulable state our patient acquired from antithrombin protein and protein S loss as part of her nephrotic range proteinuria. Since it is a rare entity, there are no specific guidelines for its management and the treatment is also not well understood. Here, we successfully treated the ophthalmic vein thrombosis with coumadin with a goal INR between 2 and 3. While the patient developed hemorrhagic cystitis, we successfully continued the anticoagulation therapy while switching the cyclophosphamide to Mycophenolate Mofetil. Orbital imaging with MRI is the gold standard for the diagnosis of this problem [1]. SOVT is commonly caused by orbital infection including sinusitis, cellulitis, vascular malformation, thyroid dysfunction, sarcoidosis, inflammatory orbital syndrome, and Wegener's granulomatosis. In rare instances, antiphospholipid syndrome has also been shown to cause SOVT. There is a case report of bilateral SOV thrombosis secondary to antiphospholipid syndrome in a female with good vision along with associated disc swelling and proptosis [2]. Sambhav et al. [3] recently reported a case of a female who developed bilateral consecutive SOVT as a presenting feature of SLE. Thrombotic events are recognized complications of nephrotic syndrome due to antithrombin protein and protein S loss, resulting in hypercoagulable state [4]. However, what makes our case remarkable is that nephrotic syndrome with class IV lupus nephritis in the absence of anti-phospholipid and anti-cardiolipin antibodies has almost never been implicated in the formation of SOVT causing unilateral blindness.

## 4. Conclusion

Acute unilateral blindness from nephrotic range proteinuria due to class IV lupus nephritis in the absence of antiphospholipid syndrome is an exceptionally rare phenomenon. To our knowledge, there is very limited management guidelines available in the literature. Our unique case presentation and successful management add to the sparse literature available on this topic. If left untreated, SOVT can lead to permanent blindness which is why further research is needed to better understand this rare phenomenon and to formulate treatment guidelines.
